# Supplementary material for: Health Experiences of African American Mothers, Wellness in the Postpartum Period and Beyond (HEAL): A Qualitative Study Applying a Critical Race Feminist Theoretical Framework
Source: Int J Environ Res Public Health. 2023 Jul 3;20(13):6283. doi: 10.3390/ijerph20136283 (PMC10341853; doi:10.3390/ijerph20136283)
Supplement: Supplementary file 1 [file ijerph-20-06283-s001.zip › ijerph-2350433-supplementary.pdf]

**Racialization:** the processes by which a group of people is defined by the "social interpretation of how [they] look", their race[1]

**Racism:** an "organized system within societies that cause avoidable and avoidable and unfair inequalities in power, resources, capacities, and opportunities across racial or ethnic groups."[2] Racism operates at institutional and individual levels to compromise health outcomes.[1,3,4]

**Institutional level racism**: reflects the fabric of racism woven deeply into various sectors (e.g., healthcare: Black-serving birthing hospitals have a lower quality of care ratings than White-serving birthing hospitals[5,6]), policies (e.g., Slavery, Jim Crow, discriminatory home lending practices, and residential segregation[7]) and programs (e.g., programs promoting unauthorized sterilization of Black women and conditioning social benefit release on sterilization[8]) in the United States.

**Individual-level racism:** the interpersonal experiences of discrimination between individuals (i.e.,., substandard care and dismissal of health concerns because of race) [9,10] and the intrapersonal experiences of discrimination within an individual (i.e.,., internalized racism; or the acceptance of messages of inferiority imposed by dominant racial groups and resultant self-devaluation[1]).

**Structural racism**: the totality of ways in which societies foster racial discrimination, through mutually reinforcing inequitable systems, that in turn reinforce discriminatory beliefs, values, and distribution of resources, which together affect the risk of adverse health outcomes. [3]

**Structural racism and discrimination:** a term coined by The National Institutes of Health to describe the "macro level conditions that limit opportunities, resources, and well-being of less privileged groups… on the basis of race/ethnicity and or other status [e.g., gender, gender identity, socioeconomic position]"; and contend that these conditions must be interrogated beyond individual experiences of discrimination that have been the traditional focus of health disparities research.[11]

**Critical Race Theory:** a "body of work that seeks to understand—and change—the relationship between race, racism, and power."[12,13] Core tenants of CRT include 1) Race is a socially constructed label used to group people into subordinate and dominant groups, thus creating an organizing hierarchy in society 2) Racism is ordinary and ubiquitous in society 3) Racism is an unescapable force that is centrally present in the lives of people of color 4) Racism is simultaneously experienced with other forms of oppression 5) an imperative to "center at the margins" : centering and privileging of voices, stories, and ideas of those historically marginalized and oppressed[14] 6) Acknowledgement of structural determinism: system or institutional level factors are key drivers of inequities. 7) Counter-narratives are a tool to "challenge dominant narratives" about a problem.[13,15] 8) Critical race theory goes beyond an academic endeavor; it demands action—a commitment to racial justice and the eradication of racial inequity.[16]

**Home Visiting:** Home visiting programs range in the services they provide, but in general provide in-home support that consists of education, caregiver skills training, counseling, screening and assessment of health, and referral to social resources for pregnant and postpartum women and their infants. Nationally there are twenty evidence-based home visiting models, ten of which are associated with improved maternal and child health outcomes (e.g., maternal health, child maltreatment and parenting practices).[17,18]

**Figure S1. Key Definitions and Concepts.**

**Table S1. Themes, Subthemes, and Representative Quotes related to Research Question 1.**

| Theme | Subtheme | Representative quotes |
|---|---|---|
| **Enduring Influence of Structural Racism** | "The layers of stress never stop": Social determinants of health influences health and care-seeking. | **Direct barrier**:<br><br>"I, personally, think about the trip and feel like I just don't want to do it. I think about, 'Dang, I got to catch this many buses or I've got to pay this much to get there?' I'm just not going. So, definitely, transportation is one of the biggest things for me for why I don't go [to healthcare appointments]. And after I been up all day at work, when I'm off work, I like to just relax. So, especially, now, with having a newborn, I need sleep. And not only that, I have [other] kids, I have a toddler." **P9** (Age 28; 1-3 months postpartum; preconception obesity)<br><br>Indirect barrier:<br><br>"I have **ongoing domestic issues** with my son's father. I had a protective order that expired last year. I changed the locks. The kids are scared that he might come back. And it just stresses me out that I am in a position where **I'm the mom and I'm their first line of defense and I feel like I can't keep them safe because they don't feel safe where we live**. And so **until I fix that, I'm not going to be okay**…And **I want to move but the housing is so hard** to find anyway because I'm in subsidy housing, I don't pay regular rent, so it's not like I can just be anywhere. It has to be somewhere I can afford. But then that is also stressful because **the subsidy housing, it's like the maintenance is not good** and so it's raggedy. There is a **hole in my bathroom ceiling** that's been there for months before COVID even happened and then it's like **mouse holes** in certain areas in the baseboard and it's been holes in the walls and I didn't have access to use my kitchen sink for days at a time…. And that's stressful enough and then you feel like you **have to stay in stuff like this because you can't afford your own housing**. So, all of that in itself is another whole layer of something else. It's like the **layers of stress never stop**. And then being stressed really can bring the lupus out. I can't afford to have a lupus attack because I have no one to take care of my kids. So, I don't have the time to take care of myself too too **mu**ch. And so that makes a difference in my health too because I don't really have the time to take care of myself because I have so much to worry about as it is…-**P6** ( Age 35; 6-9 months postpartum; preconception chronic hypertension, obesity, preeclampsia in pregnancy):<br><br>"Baltimore's a violent city. No matter where you go, it's something that's always happening…If I walk out the house to the left and to the right and I go about two blocks, there's drug dealers on both sides. And it just worries me that I have my children with me because-- a lot of times, [in our neighborhood] you might see like a mom and her kids and the little four-year-old got shot or the three-year-old got shot or the baby got—I worry. I'm kind of nervous that if I take my baby outside and they start shooting and then we're outside and we're stuck. And by me also just having a newborn, I have a stroller. So, it's like I can't cover two kids at one time"- **P19** (33 years old; 1-3 months postpartum; preconception obesity, preeclampsia): |
| **Personally Mediated Racism in Healthcare and Beyond** | "They Judge Us Before They Know Us": Impact of Gendered Racism on the Health Care Encounter | "I think the way they treated me had something to do with me being Black and the fact that-- well, I get social services. Because they know things like that. So, yeah, I guess you could say that where I live at, things like that. I feel like all that plays a factor in how healthcare providers and certain people look at us. They think because of where I'm from or what my situation may be that I don't take care of myself or I'm this and I'm that? So, I feel like they judge us before they get to know us."- **P8** (Age 28; 6-9 months postpartum; preconception obesity, preeclampsia)<br><br>"I feel like sometimes they know who has a support system, and who doesn't have a support system. And for the most part, they start off with the belief Black people, they don't have anybody. We're single moms. We're coming here unsupported. they think that they can treat you a certain way less than the standard."- **P12**: (24 years old; 3-6 months postpartum; preconception obesity): |
| | "They Treated Me Like an Inmate": Obstetric Racism in the Perinatal Period | "I kept saying I feel like I am dying. And [the nurse] was like, '"It's not that bad. You're not dying. You're just being a little overdramatic' … when the doctor left and the nurse left, I had to break down to my mother like, ' I don't have it in me. They're gonna kill me'. Because I felt like |

| | | |
|---|---|---|
| | | that's [what] was going to happen anyway cuz I was being experimented on. I felt like nobody was taking what I was saying at face value."- **P15:** (Age 28; 0-1 months postpartum; preconception obesity; gestational diabetes and preeclampsia)<br><br>**"**I just felt like I was slipping away and that nobody was paying any mind. I felt like I just wanted to give up. And they were saying like, 'It's going to be okay.'I felt like nobody was listening. I kept saying the epidural doesn't work. I felt like if somebody listened, then maybe I wouldn't have lost so much blood because I was fighting the [scalpel]-- when you're getting cut, it hurts."- **P15:** (Age 28; 0-1 months postpartum; preconception obesity; gestational diabetes and preeclampsia)<br><br>"I think it had something to do with me being Black… I kept trying to get me ibuprofen, and I kept telling them, 'Miss, can you please go look at my records? I'm a bariatric patient, I cannot have anything with ibuprofen in it, and she thought I was lying. So they wouldn't give me Percocet or whatever. I had to basically cry for the head nurse to go look at my chart to see that I was a bariatrics patient, so I wasn't lying about not being able to take that ibuprofen. They thinking I wanted to get high off the medication."- **P4** (36 years old; 9-12 months postpartum; preconception chronic hypertension and preeclampsia):<br><br>" I think that all Black women should just have their babies at home in a jacuzzi or] instead of in a hospital because I just--I just think something's always going to happen that's bad."- **P19** (33 years old; 1-3 months postpartum; preconception obesity, preeclampsia ): |
| | "It's Scary Being a Black Mother to Black Kids": Vicarious Racism and Hypervigilance | "With things in the news: watching young Black men being killed for no reason… that's something that always sat in the back of my head once I found out I was having a boy, to just protect him as he gets older. To keep him in a safe space especially living somewhere like Baltimore which you and I both know it's not the safest place per se"- **P16** (Age 34; 1-3 months postpartum; preconception obesity and chronic hypertension):<br><br>"That's for any Black woman, especially if raising boys. I feel like you can't--let your guard down, let your kids be kids, and let them explore and have open minds to different things. Black women have to hover over their boys so much [because] they don't want anything to happen…. which can be an up-and-down thing and can bring a lot of stress onto a woman."- **P9** (Age 28; 1-3 months postpartum; preconception obesity |
| **Sociocultural Beliefs About Health and Healthcare** | "Instilled in Me": Cultural, Social, and Familial Ideals About Healthcare | "I know my mother, she had this little knot on her hand, and she would complain here and there to me about it. She was telling her doctor about it, but he would never do nothing about it. And one day she transferred doctors. They had to schedule her an emergency appointment. It was a cancer, this knot in her hand and it had been there for years…. And when I was about seven or eight, I really had a breathing problem. It had gotten so out of control. And when I first started telling [the doctor] about the breathing problem, they didn't take it as serious. My mother was taking me to the hospital, and they would put me on a nebulizer, but [the doctors] would say I didn't have a breathing problem. Both times, I think it had something to do with me being Black, and I think it had something to do with my mother being Black."." - **P4** (36 years old; 9-12 months postpartum; preconception chronic hypertension and preeclampsia):<br><br>"My mother is very--go to the doctors. We always [went to the] eye doctor, the dentist, the primary care doctor. That was something that we were big on in our house. So, I guess that has been instilled in me."- **P7** (Age 38; 1-3 months postpartum; gestational diabetes and preeclampsia in pregnancy): |
| | " [Just] Pray it Away": Mental Health Stigma and Utilization | "But I do feel like it's very important especially within the Black community because you always want to go pray something away or no one want to talk about their mental health…. [Or} sometimes people don't have anybody to ask for help and that's when things get bad."-**P7** (Age 38; 1-3 months postpartum; gestational diabetes and preeclampsia in pregnancy):<br><br>"In the Black community, mental health and things like that, they're frowned upon. If you're going to see a psychiatrist or a therapist, it's like, 'Oh, are you going to see a shrink?' They kind of look down on you."- **P14** (32years old; 1-3 months postpartum; gestational diabetes and preeclampsia):<br><br>But I think mental health needs to be more readily accessible to people because it's very hard. Insurance doesn't want to pay for certain things. And insurance will pay for medical but as far as therapy, your copays…And then if you're on medical assistance, it can be even more difficult. And I think mental health needs to be more readily available to everybody. Period. You shouldn't have to pay arm and a leg if you want to go see a therapist. If you want to talk about your issues and go see therapy if just taking the pill doesn't work. Mental health, I feel like |

| | | |
|---|---|---|
| | | it's a need. It's just I don't understand why it's not as available as cardiology."- **P7** (Age 38; 1-3 months postpartum; gestational diabetes and preeclampsia in pregnancy): |
| | "I am here, I am a person": Counterstereotypes and Faith Foster resilience and Agency | " I am telling you, I survived the story of my life. I probably make it look easy, I don't complain a lot. I still smile, I still help everybody else around me as much as I can, but I still have so much going on that people wouldn't even know…But I'm doing it every day… I feel this is a system flaw. It's that way. It's always been that way. We're not really supported at all. It's like we have to fight for our support. We have to [be] like, 'Hello. I'm here. I'm a person.'"- **P6** (35 years old; 6-9 months postpartum; preconception chronic hypertension, obesity, preeclampsia):<br><br>"I have dealt with tragedies. I'm not saying that I haven't, because me and my husband, we lost our oldest son to gun violence two years ago—right around the corner from our house… But yeah-- I mean, this is where you're at, where you live in your life, how the world is, how things are happening. I mean, we just don't kind of like just get all in a negative, oppressive mood about things like that, you know? When [I] stay deeply rooted in the positive and my religion and my faith…every day, I just wake up blessed." **P18** (Age 43; 0-1months postpartum; preconception obesity and chronic hypertension; gestational diabetes and preeclampsia<br><br>"I know sometimes it can be rough as a Black woman because I feel like we get the least respect when it comes to outsiders and stuff. But I don't worry about that… I just try to stay positive as I can and around positive people."- **P8** (28 years old; 6-9 months postpartum; preconception obesity, preeclampsia)<br><br>"He wasn't listening to me, so I changed doctors. There comes a time that you can't let that stop you…if someone isn't listening to you or you don't like that doctor, you can't just stop and say you're not going to go get the situation handled. You got to keep going, and you got to find someone else who will listen."-**P18** (Age 43; 0-1months postpartum; preconception obesity and chronic hypertension; preeclampsia and gestational diabetes) |

**Table S2. Themes, Subthemes, and Representative Quotes related to Research Question 2.**

| Theme | Subtheme | Representative quotes |
|---|---|---|
| **Barriers to Postpartum Care Transitions** | "I Didn't Think I Needed to See My Primary Care Doctor:"<br><br>Limited Patient Knowledge about Postpartum Health Needs Following Cardiometabolic Complications of Pregnancy and Unique Role of Primary Care Providers | "I mean [the obstetric providers] told me the basics. They told me [about] preeclampsia—[but] honestly, even during my pregnancy I did not know the difference between someone who had chronic hypertension and preeclampsia…"- **P17** (38 years old; 0-1 months postpartum; preconception chronic hypertension and preeclampsia):<br><br>"I thought you could only be preeclamptic during pregnancy. I didn't know I could still develop preeclampsia six weeks after [pregnancy]. That's why I just thought it was normal swelling the whole time. I didn't respond to it because they told me it was normal swelling."- **P18** (Age 43; 0-1 months postpartum; preconception obesity and chronic hypertension; preeclampsia and gestational diabetes in pregnancy):<br><br>"They was giving me insulin shots for sugar [ during pregnancy], but they didn't send me home on none of that. They said that the sugars were normal and …I don't need any insulin. I guess she said the diabetes is…under control?"- **P15** (35 years old; 0-1months postpartum; preconception obesity; preeclampsia and gestational diabetes):<br><br>"I haven't seen my primary care doctor in a long time… because my GYN doctor did the things that needed to be done."- **P19** (33 years old; 1-3 months postpartum; preconception obesity, preeclampsia): |
| | Lack of "Information Sharing" Between Obstetric and Primary Care Providers | "I'm like I want to feel comfortable that doctors are communicating and sharing information to make my care …. That OB/GYN doctor, she was like, 'Well, why is your rheumatologist referring you to get an ultrasound? He just needs to just worry about your lupus and let us take care of the baby part'. But my rheumatologist said, 'You're my patient with lupus, and you're pregnant. I do have to worry about you because you have lupus at all times, and you're my patient, so I have to refer you to do certain things…"- **P6** (35 years old; 6-9 months postpartum; preconception chronic hypertension, obesity, preeclampsia): |
| **Facilitator of Postpartum Care Transitions: Patient-Provider Relationships** | "Not Just Another Chart": Patient-Centered Care, Humanistic care and Individuation improved Experiences of Care | "He [ primary care provider] knows me. He really remembers, knows what we talked about last time... He makes me feel like he's been listening [and] yeah, I'm not just another chart... He consults with me what he thinks I should do. He refers me out whenever he feels as though I need to be referred somewhere else. And if I don't like it, I feel comfortable coming to him saying, 'No, let's make another adjustment. And he's open to doing that also. So, we just work through it together."- **P18** (Age 43; 0-1 months postpartum; preconception obesity and chronic hypertension; preeclampsia and gestational diabetes): |
| | "I Keep Him in the Loop": Preconception Relationships Improve Care Continuity and Communication | "Yeah. It's a good relationship. I've been a patient of hers for I'd probably say, maybe 8 or 10 years. I did discuss [plans to get pregnant] with her last time I saw her which, and I'd asked her when I got on the blood pressure medicine was it safe to be on this specific medicine while I was pregnant and for breastfeeding…So we talked about that. And throughout the pregnancy when I found out I was pregnant and probably once or twice every trimester I would just message just to kind of keep her up to date with how the pregnancy was going."-**P1**6 (Age 34; 1-3 months postpartum; preconception obesity and chronic hypertension): |
| | "I Didn't Have to Put on a Professional Face": Racial and Gender Concordance Supports Open Communication | "I feel a little bit more comfortable dealing with other Black women because they can relate to me more. One of the doctors in the OB practice is a Black woman and I felt a lot more comfortable talking to her, because she can relate to things that I'm saying. I didn't feel like I had to put on more of the professional face if you will be talking to a White doctor versus a Black one…. And at the same time the first doctor and the only doctor that I ever had for much of my life was a White woman. And she treated me with respect. [She] took care of us."- **P14** (Age 32; 1-3 months postpartum; preeclampsia and gestational diabetes): |
| **Postpartum Health and Healthcare needs** | "Postpartum is No Joke": Lack of Preparation for Unique Mental Health and Social Support Needs that Emerge Postpartum | "And I just told [my OB] about a lot of depression and stuff that I had. And she prescribed me antidepressants again to get back on. And she's like, 'Well you should be good in two weeks.' And I'm thinking to myself, 'You think all of this is going to be undone in two weeks?' - **P17** (Age 38; 0-1 months postpartum; preconception chronic hypertension and preeclampsia):<br><br>"With young Black mothers…I think if they had more groups where they could all sit around and realize they're not the only one that's scared and stuff like that and exchange numbers. And they can call this girl when they're going through something..."- **P4** (Age 36; 9-12 months postpartum; preconception chronic hypertension and preeclampsia): |

1. Paradies Y, Ben J, Denson N, et al. Racism as a determinant of health: A systematic review and meta-analysis. PLoS One. 2015;10(9):1-48. doi:10.1371/journal.pone.0138511
2. Phyllis Jones C. Levels of Racism: A Theoretic Framework and a Gardener's Tale. Am J Public Health. 2000;90(8). Accessed December 17, 2018. https://www.ncbi.nlm.nih.gov/pmc/articles/PMC1446334/pdf/10936998.pdf
3. Bailey ZD, Krieger N, Agénor M, Graves J, Linos N, Bassett MT. Structural racism and health inequities in the USA: evidence and interventions. Lancet. 2017;389(10077):1453-1463. doi:10.1016/S0140-6736(17)30569-X
4. Ahmed AT, Mohammed SA, Williams DR. Racial discrimination & health: pathways & evidence. Indian J Med Res. 2007;126(4):318-327. Accessed December 16, 2018. http://www.ncbi.nlm.nih.gov/pubmed/18032807
5. Howell EA, Egorova NN, Balbierz A, Zeitlin J, Hebert PL. Site of delivery contribution to black-white severe maternal morbidity disparity. Am J Obstet Gynecol. 2016;215(2). doi:10.1016/j.ajog.2016.05.007
6. Creanga AA, Bateman BT, Mhyre JM, Kuklina E, Shilkrut A, Callaghan WM. Performance of racial and ethnic minority-serving hospitals on delivery-related indicators. Published online 2014. doi:10.1016/j.ajog.2014.06.006
7. Crear-Perry J, Correa-de-Araujo R, Lewis Johnson T, McLemore MR, Neilson E, Wallace M. Social and Structural Determinants of Health Inequities in Maternal Health. J Women's Heal. 2020;00(00):jwh.2020.8882. doi:10.1089/jwh.2020.8882
8. Taylor JK. Structural Racism and Maternal Health Among Black Women. J Law, Med Ethics. 2020;48(3):506-517. doi:10.1177/1073110520958875
9. Dominguez TP. Adverse Birth Outcomes in African American Women: The Social Context of Persistent Reproductive Disadvantage. Soc Work Public Health. 2011;26(1):3-16. doi:10.1080/10911350902986880
10. NPR/Propublica. Lost Mothers: Maternal Mortality In The U.S. : NPR.https://www.npr.org/series/543928389/lost-mothers. Published 2018. Accessed April 6, 2019.
11. Structural Racism and Discrimination. Accessed April 20, 2021. https://nimhd.nih.gov/resources/understanding-health-disparities/srd.html
12. Cross RI. Commentary: Can critical race theory enhance the field of public health? A student's perspective. Ethn Dis. 2018;28:267-270. doi:10.18865/ED.28.S1.267
13. Delgado RSJ. Critical Race Theory: An Introduction. New Yok University Press; 2012. doi:10.5860/choice.50-2938
14. hooks  bell. Feminist Theory : From Margin to Center.; 2014.
15. Ford CL, Airhihenbuwa CO. Commentary: Just what is critical race theory and what's it doing in a progressive field like public health? Ethn Dis. 2018;28:223-230. doi:10.18865/ed.28.S1.223
16. Ford CL. PUBLIC HEALTH CRITICAL RACE PRAXIS: AN INTRODUCTION, AN INTERVENTION, AND THREE POINTS FOR CONSIDERATION. Accessed July 27, 2021. http://www.who.int/about/mission/en/.
